# Clinical Setting Comparative Analysis of Uropathogens and Antibiotic Resistance: A Retrospective Study Spanning the Coronavirus Disease 2019 Pandemic

**DOI:** 10.1093/ofid/ofad676

**Published:** 2023-12-22

**Authors:** Alexandra M Young, Mark M Tanaka, Christopher Yuwono, Michael C Wehrhahn, Li Zhang

**Affiliations:** School of Biotechnology and Biomolecular Sciences, University of New South Wales, Sydney, New South Wales, Australia; School of Biotechnology and Biomolecular Sciences, University of New South Wales, Sydney, New South Wales, Australia; School of Biotechnology and Biomolecular Sciences, University of New South Wales, Sydney, New South Wales, Australia; Douglass Hanly Moir Pathology, a Sonic Healthcare Australia Pathology Practice, Macquarie Park, New South Wales, Australia; School of Biotechnology and Biomolecular Sciences, University of New South Wales, Sydney, New South Wales, Australia

**Keywords:** urinary tract infection, trimethoprim, uropathogens, antibiotic resistance, aged care facilities

## Abstract

**Background:**

Antimicrobial resistance (AMR) in uropathogens has been increasing in Australia. Many nations observed heightened AMR during the coronavirus disease 2019 (COVID-19) pandemic, but it is not known how this may vary across clinical settings and in nations with lower infection rates.

**Methods:**

We investigated the uropathogen composition and corresponding antibiotic resistance of 775 559 Australian isolates from the community, hospitals, and aged care facilities before (2016–2019) and during (2020–2022) the COVID-19 pandemic. A mathematical model was developed to predict the likelihood of resistance to currently recommended antibiotics for treating urinary tract infections (UTIs).

**Results:**

Among uropathogens originating from the community, hospitals, and aged care facilities, *Escherichia coli* accounted for 71.4%, 57.6%, and 65.2%, respectively. During the COVID-19 pandemic period, there was an increase in UTIs caused by *E coli* across all settings. Uropathogens from aged care and hospitals frequently showed higher resistance to antibiotics compared to those isolated from the community. Interestingly, AMR among uropathogens showed a declining trend during the COVID-19 pandemic. Based on the resistance patterns of the past 3 years, our modeling predicted that 30%, 42.6%, and 38.8% of UTIs in the community, hospitals, and aged care facilities, respectively, would exhibit resistance to trimethoprim treatment as empirical therapy. In contrast, resistance to nitrofurantoin was predicted to be 14.6%, 26%, and 24.1% from these 3 respective settings.

**Conclusions:**

Empirical therapy of UTIs in Australia with trimethoprim requires evaluation due to high rates of resistance observed across clinical settings.

## Teaser

High rates of trimethoprim resistance among Australian uropathogens were observed across clinical settings, with mathematical modeling supporting a change to the empirical therapy for urinary tract infection. Altered resistance and uropathogen composition were identified associated with the COVID-19 pandemic period.

Urinary tract infections (UTIs), predominately manifesting as cystitis, are highly prevalent, with an estimated global incidence of >400 million cases per year [1]. In Australia, UTIs account for approximately 1.5% of all general practitioner consultations [2, 3]. The recommended antibiotics for empirical treatment of UTIs in Australia includes trimethoprim, nitrofurantoin, cefalexin, and amoxicillin with clavulanate [4]. There have been reports of increasing local rates of antibiotic resistant uropathogens in Australia [5–8]. However, limited published data are available regarding the uropathogen composition among patients from diverse healthcare settings and the concurrent patterns of antibiotic resistance and the impact this has on treatment recommendations.

Recent investigations from other nations have reported an increase in antimicrobial resistance (AMR) during the coronavirus disease 2019 (COVID-19) pandemic [9–16]. These studies, primarily from regions recording high rates of COVID-19 infection and disease burden, predominately focused on the early stages of the pandemic. There are limited comparable investigations conducted in areas with lower rates of COVID-19 infection such as Australia. Few studies conducted within Australian hospitals throughout 2020 observed stable or decreased presence of multidrug-resistant organisms [17, 18]. However, Australian studies have not examined if the enhanced infection control and antibiotic stewardship measures continued to protect against AMR as COVID-19 cases and hospitalizations surged throughout 2021–2022, with uropathogen composition also unknown.

In this study we investigated the bacterial species composition of uropathogens and their antibiotic resistance within 3 settings including the community, hospitals, and aged care facilities. Our analysis was conducted on a large population from New South Wales, Australia, from 2016 to 2022. We additionally compared uropathogens and their antibiotic resistance during the COVID-19 pandemic and the pre-COVID-19 period. Furthermore, we developed a mathematical model to predict the resistance of uropathogens to the recommended antibiotics for empirical treatment of UTIs in Australia. Our data provide valuable insights for medical professionals in effectively managing UTIs and for the relevant Australian authorities in formulating antibiotic treatment approaches of UTIs.

## METHODS

### Microbiological Data

The data analyzed in this study were provided by the Douglass Hanly Moir (DHM) pathology diagnostic laboratory, which included the identities of uropathogens isolated between 2016 and 2022 and their corresponding antibiotic susceptibility. The provided data contained collated bacterial isolations and corresponding sensitivities within each of the 3 clinical settings of the community, hospitals, and aged care facilities. The clinical samples were from the state of New South Wales, Australia (Supplementary Figure 1). Only the first isolate of a given species per person per year was included for analysis. Significant growth thresholds were reported depending on patient age, urine sample collection method, and white blood cell count. Bacterial isolates were identified depending on a combination of biochemical tests, growth and appearance on Mueller-Hinton and Brilliance UTI agar, and matrix-assisted laser desorption/ionization–time-of-flight mass spectrometry. The disk diffusion method via calibrated dichotomous sensitivities was used for antibiotic sensitivity testing for all isolates with the exception of *Pseudomonas* spp, which were run on Vitek2 (broth microdilution). Minimum inhibitory concentrations were based on European Committee on Antimicrobial Susceptibility Testing guidelines from 2018, replacing the Clinical and Laboratory Standards Institute methodology used in 2016–2017.

### Data Analysis and Statistical Methods

A χ^2^ test of independence was completed using adjusted residuals to evaluate the relationship between clinical settings and bacterial isolate incidence as well as to assess differences in species incidence preceding (2016–2019) and during (2020–2022) the pandemic period. The strength of association between clinical settings and isolate incidence was assessed using Cramer's V. Resistance rates for bacterial isolates within each clinical setting were analyzed for change over time using the Cochran-Armitage test, noting any changes in trend between the years proceeding and during the pandemic. The Cochran-Mantel-Haenszel test was applied to assess potential differences in resistance rates between clinical settings while controlling for year.

The Bonferroni correction was applied to Cochran-Mantel-Haenszel and χ^2^ tests to adjust for multiple comparisons. Statistical significance before corrections was defined as *P* < .05 for all tests. Statistical analyses were completed using R software (via RStudio v1.4.1717).

### Probability Model for Prediction of Uropathogen Antibiotic Resistance

We estimated the probability that an infection is resistant to recommended antibiotics for empirical treatment of uncomplicated UTI as follows. Let *U* be the set of bacterial pathogens known to cause UTIs. If *rij* is the probability of resistance to drug *i* given infection with pathogen *j*, and *pj* is the probability of infection with pathogen *j*, the probability that the patient is resistant to treatment *i* is:


$$P_{i}=\underset{j\in U}{\sum }\;r_{ij}p_{j}.$$

We estimate *pj* with $f_{j}/\underset{u\in U}{\sum }\;f_{u}$ where *fj* is the frequency of pathogen *j* among UTIs, and we estimate *rij* with observed antibiotic resistance frequencies.

To study the effect of the pre-COVID-19 and COVID-19 periods, we used data over the years 2016–2019 and 2020–2022, respectively. To study the effect of setting on the probability of treatment sensitivity/resistance, we used frequency data within the settings of the community, hospitals, and aged care facilities.

## RESULTS

During the period spanning from 2016 to 2022, a total of 1 183 298 bacterial pathogens were isolated from the clinical samples processed at DHM pathology. Bacterial uropathogens predominated (66% [n = 775 559]), with fewer isolates from blood (<1% [n = 4 736]) and other specimen sources (34% [n = 403 003]).

Among the uropathogens, the largest proportion originated from the community, accounting for 83% (n = 644 510), followed by aged care at 10% (n = 74 011) and hospital isolates (7% [n = 57 038]).

### Composition of Primary Bacterial Uropathogens and Clinical Setting of Isolation

Approximately 90% of bacterial isolates were attributed to 5 pathogen groups, which was consistently observed over the 7 years from 2016 to 2022 (Table 1). *Escherichia coli* was most prevalent, accounting for >60% of the isolated pathogens, followed by *Enterococcus*, *Klebsiella*, *Pseudomonas*, and *Enterobacter* spp (Table 1). *Staphylococcus saprophyticus* was additionally reported during 2021 and 2022. All other organisms were combined into a single category for further analysis, including *Streptococcus*, *Morganella*, and *Citrobacter* spp (Supplementary Table 1).

**Table 1.** Isolation of Uropathogens From Patients With Urinary Tract Infection, 2016–2022

Organism Group	No. of Isolates for Individual Uropathogen (% of Total Yearly Uropathogen Isolates)							
	2016	2017	2018	2019	2020	2021	2022	Total
*Escherichia coli*	75 483 (57.48)	75 754 (58.27)	70 326 (64.52)	70 364 (64.97)	66 211 (70.02)	65 068 (66.97)	73 268 (69.63)	496 474 (64.014)
*Enterococcus* spp	19 624 (14.94)	18 037 (13.87)	11 527 (10.58)	10 996 (10.15)	10 854 (11.48)	11 515 (11.85)	10 577 (10.05)	93 130 (12.01)
*Klebsiella* spp	9124 (6.95)	9270 (7.13)	8715 (8.00)	9831 (9.08)	9601 (10.15)	9566 (9.85)	11 419 (10.85)	67 526 (8.71)
*Pseudomonas* spp	4445 (3.38)	4516 (3.47)	4251 (3.90)	4357 (4.02)	4502 (4.76)	4204 (4.33)	3492 (3.32)	29 767 (3.84)
*Enterobacter* spp	2821 (2.15)	2844 (2.19)	2894 (2.66)	1716 (1.58)	1834 (1.94)	1710 (1.76)	1912 (1.82)	15 731 (2.03)
*Proteus* spp	5444 (4.15)	5447 (4.19)	NA	NA	NA	NA	NA	10 891 (1.40)
*Staphylococcus saprophyticus*	NA	NA	NA	NA	NA	3665 (3.77)	3535 (3.36)	7200 (0.93)
All other isolates	14 380 (10.95)	14 138 (10.87)	11 278 (10.35)	11 037 (10.19)	1558 (1.65)	1425 (1.47)	1024 (0.97)	54 840 (7.07)

All samples were urine samples. For all settings, the 4 most abundant organisms were *Escherichia coli*, *Enterococcus* spp, *Klebsiella* spp, and *Pseudomonas* spp. Data for *Proteus* spp were only available for 2016–2017, and only 2021–2022 for *Staphylococcus saprophyticus*. Species composition of all other isolates category is available in Supplementary Table 1.

Abbreviation: NA, not available.

In 2022, *E coli* and *S saprophyticus* were significantly more common in samples from the community (71.4% vs 4.1%, respectively) compared to those from hospitals (57.6% vs 0.5%) and aged care facilities (65.2% vs 0%; Table 2; adjusted α = .0012, *P* < .0001). *Klebsiella*, *Pseudomonas*, and *Enterobacter* spp were present significantly more often within samples from hospitals and aged care facilities. *Enterococcus* isolation had a significantly higher prevalence in samples from hospitals (*P* < .001; Table 2). Consistent results were obtained from analysis of the entire 7-year period from 2016 to 2022 (Supplementary Table 2).

**Table 2.** χ^2^ Comparison of Uropathogen Isolation During 2022 From Patients With Urinary Tract Infection, by Clinical Setting

Organism	Setting		
	Community, No. (%)	Hospital, No. (%)	Aged Care, No. (%)
*Escherichia coli*	61 276 (71.4)	4765 (57.6)	7227 (65.2)
	[25.6]	[−24.8]	[−10.6]
*Enterococcus* spp	8351 (9.7)	1277 (15.4)	949 (8.6)
	[−7.4]	[17]	[−5.5]
*Klebsiella* spp	8622 (10)	1140 (13.8)	1657 (15)
	[−17.8]	[8.9]	[14.7]
*Pseudomonas* spp	2051 (2.4)	689 (8.3)	752 (6.8)
	[−35.5]	[26.5]	[21.6]
*Enterobacter* spp	1332 (1.6)	259 (3.1)	321 (2.9)
	[−13.6]	[9.3]	[9]
*Staphylococcus saprophyticus*	3492 (4.1)	43 (0.5)	0 (0)
	[26.8]	[−14.9]	[−20.7]
All other isolates	753 (0.9)	101 (1.2)	170 (1.5)
	[−6.7]	[2.4]*	[6.4]
χ^2^(12)	2984		
Cramer's V	0.119 (95% CI, .115–.123)		

Adjusted standardized residuals appear in square brackets below the isolation frequencies. After Bonferroni correction for multiple comparisons α = .0012. All adjusted residuals were individually consistent with a significant association between clinical setting and incidence of species isolation (*P* < .0001), except *P > .05.

Abbreviation: CI, confidence interval.

### Changes in Uropathogen Composition During the COVID-19 Pandemic Years

The community, hospitals, and aged care facilities each had significantly altered species composition when comparing the COVID-19 pandemic years (2020–2022) to the pre-COVID-19 (2016–2019) years (adjusted α = .005; all settings χ^2^: *P* < .001; Table 3). When considering the relative isolation incidence of the 5 most frequently obtained uropathogens, *E coli* significantly increased during the pandemic period (community, 2.0%, hospitals, 2.6%, aged care, 1.9%; all adjusted residuals *P* < .0001).

**Table 3.** χ^2^ Comparison of Uropathogen Isolation by Clinical Setting Before (2016–2019) and During (2020–2022) the Pandemic Period

Organism	Community			Hospital			Aged Care		
	2016–2019, No. (%)	2020–2022, No. (%)	Net Change	2016–2019, No. (%)	2020–2022, No. (%)	Net Change	2016–2019, No. (%)	2020–2022, No. (%)	Net Change
*Escherichia coli*	254 499 (72.1)	172 496 (74.1)	↑2.0%**	15 913 (53.1)	11 938 (55.7)	↑2.6%**	21 515 (61.7)	20 113 (63.6)	↑1.9%**
	[−16.71**]	[16.71**]		[−5.82**]	[5.82**]		[−5.1**]	[5.1**]	
*Enterococcus* spp	49 228 (14.0)	25 674 (11.0)	↓3.0%**	6357 (21.2)	3735 (17.4)	↓3.8%**	4599 (13.2)	3537 (11.2)	↓2.0%**
	[32.76**]	[−32.76**]		[10.66**]	[−10.66**]		[7.9**]	[−7.9**]	
*Klebsiella* spp	29 719 (8.4)	23 315 (10.0)	↑1.6%**	3198 (10.7)	2823 (13.2)	↑2.5%**	4023 (11.5)	4448 (14.1)	↑2.5%**
	[−20.80**]	[20.80**]		[−8.69**]	[8.69**]		[−9.8**]	[9.8**]	
*Pseudomonas* spp	10 862 (3.1)	7405 (3.2)	↑0.1%	3221 (10.7)	2214 (10.2)	↓0.5%	3486 (10.0)	2579 (8.2)	↓1.8%**
	[−2.22]	[2.22]		[1.52]	[−1.52]		[8.2**]	[−8.2**]	
*Enterobacter* spp	8412 (2.4)	3784 (1.6)	↓0.8%**	1302 (4.3)	741 (3.5)	↓0.8%**	1238 (3.6)	931 (2.9)	↓0.6%*
	[19.89**]	[−19.89**]		[5.08**]	[−5.08**]		[4.4*]	[−4.4*]	
χ^2^(4)	1796.9	…	…	200.3	…	…	227.6	…	…
Cramer's V	0.055	(95% CI, .053–.058)		0.062	(95% CI, .053–.07)		0.058	(95% CI, .050–.066)	

Adjusted standardized residuals appear in square brackets below the isolation frequencies. After Bonferroni correction for multiple comparisons: α = .005.

Abbreviation: CI, confidence interval.

*P < .005; **P < .0001.

Within the community, during the pandemic period an increased incidence of *Klebsiella* (2.9%) and a reduced incidence of *Enterococcus* (3.0%) and *Enterobacter* (0.8%) were observed (all adjusted residuals *P* < .001). The changes detected within the hospital setting were consistent. Within aged care, the statistically significant adjusted residuals similarly indicated a reduced incidence of *Enterococcus* (2.0%) and *Enterobacter* (0.6%) following the beginning of the pandemic (Table 3). The aged care setting additionally recorded a statistically significant reduction in *Pseudomonas* spp (1.8%).

### Antibiotic Resistance Patterns of Uropathogens

Analysis was conducted of the antibiotic resistance patterns of the 6 bacterial species, which constituted >90% of uropathogens within our data. Antibiotics recommended for empirical treatment of uncomplicated UTI within Australia were trimethoprim, nitrofurantoin, cefalexin, and amoxicillin with clavulanate (Supplementary Table 3).

Resistance was expressed as a percentage of the number of isolates tested for a given antibiotic within the community, hospitals, or aged care facilities (Supplementary Table 3). For cases where <90% of species isolates were tested for a given antibiotic, resistance was instead calculated using the total number of isolates obtained. Where resistance data were not available for an antibiotic for each of the 7 years, it was excluded from analysis (Supplementary Figures 2 and 3), with the exception of *S saprophyticus* due to its prevalence as a common uropathogen in young women [19].

#### Escherichia coli

**Figure 1.**
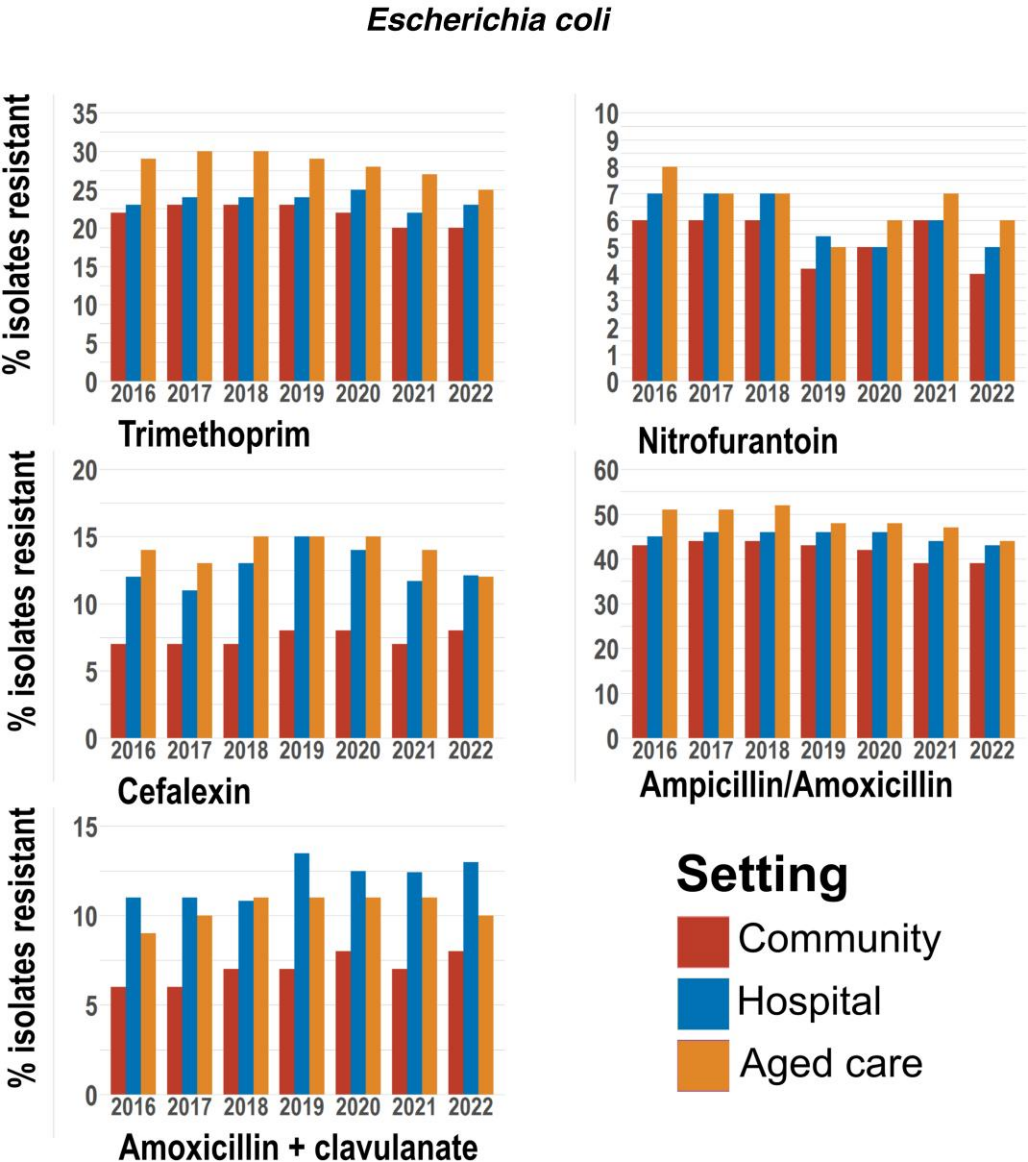
Resistance of *Escherichia coli* to antibiotics over time across clinical settings. Resistance is expressed as a percentage of the number of isolates tested within each setting, which may not represent the total number obtained. The counts for total individual species isolates obtained and the number tested for each antibiotic are shown in Supplementary Table 4.

*Escherichia coli* strains isolated from aged care facilities generally recorded the highest rates of antibiotic resistance, followed by isolates from hospital samples (Figure 1). The exception was amoxicillin with clavulanate, where resistance was highest in hospitals followed by aged care and the community (Figure 1, Table 4). Up to 30% of isolates from aged care facilities were resistant to trimethoprim, with the odds of resistance 1.27 times higher within aged care compared to hospitals, and 1.41 times higher than the community (Table 4). Cefalexin resistance was lowest within the community, with the odds of resistance 2.01 times higher in aged care and 1.80 times higher in hospitals. Nitrofurantoin resistance remained below 10% in *E coli* isolates from all settings.

**Table 4.** Uropathogen Antibiotic Resistance Odds Ratio, by Clinical Setting

Organism	Antibiotic	Setting	Odds Ratio	(95% CI)
*Escherichia coli*	Trimethoprim	Aged care: Community	1.41**	(1.37–1.44)
		Aged care: Hospital	1.27**	(1.23–1.32)
		Hospital: Community	1.10**	(1.07–1.14)
	Nitrofurantoin	Aged care: Community	1.26**	(1.21–1.31)
		Aged care: Hospital	1.10**	(1.03–1.17)
		Hospital: Community	1.15**	(1.10–1.22)
	Cefalexin	Aged care: Community	2.01**	(1.95–2.07)
		Aged care: Hospital	1.12**	(1.07–1.17)
		Hospital: Community	1.80**	(1.73–1.87)
	Ampicillin/amoxicillin	Aged care: Community	1.31**	(1.28–1.34)
		Aged care: Hospital	1.15**	(1.12–1.19)
		Hospital: Community	1.14**	(1.11–1.16)
	Amoxicillin + clavulanate	Hospital: Aged care	1.18**	(1.12–1.24)
		Hospital: Community	1.82**	(1.75–1.89)
		Aged care: Community	1.54**	(1.48–1.59)
*Enterococcus* spp	Nitrofurantoin	Hospital: Aged care	1.13**	(1.01–1.26)
		Hospital: Community	6.07**	(5.54–6.66)
		Aged care: Community	5.19**	(4.69–5.75)
	Ampicillin/amoxicillin	Hospital: Aged care	NS	
		Hospital: Community	5.39**	(4.98–5.84)
		Aged care: Community	5.12**	(4.70–5.58)
	Vancomycin	Hospital: Aged care	1.20**	(1.05–1.37)
		Hospital: Community	6.26**	(5.61–6.99)
		Aged care: Community	5.10**	(4.50–5.77)
*Klebsiella* spp	Trimethoprim	Aged care: Community	1.57**	(1.47–1.68)
		Aged care: Hospital	1.28**	(1.16–1.41)
		Hospital: Community	1.24**	(1.14–1.35)
*Enterobacter* spp	Trimethoprim	Aged care: Community	2.22**	(1.97–2.50)
		Aged care: Hospital	NS	
		Hospital: Community	1.87**	(1.64–2.12)

Odds ratios were calculated using the Cochran-Mantel-Haenszel test. After Bonferroni correction for multiple comparisons: α = .0167.

Abbreviations: CI, confidence interval; NS, not statistically significant.

*P < .01; **P < .001.

Resistance to trimethoprim in aged care and community settings showed a decreased trend during the pandemic years (*P* < .001), whereas cefalexin resistance was found to increase within the prepandemic period (community and hospital *P* < .001, aged care *P* = .009) before decreasing between 2019 and 2022 (aged care and hospital *P* < .001; community *P* = .03).

Resistance to ampicillin/amoxicillin was consistently between 40% and 50% and decreased across the 7-year period within all settings (community and aged care *P* < .001, hospital *P* = .004).

#### *Enterococcus* Species

**Figure 2.**
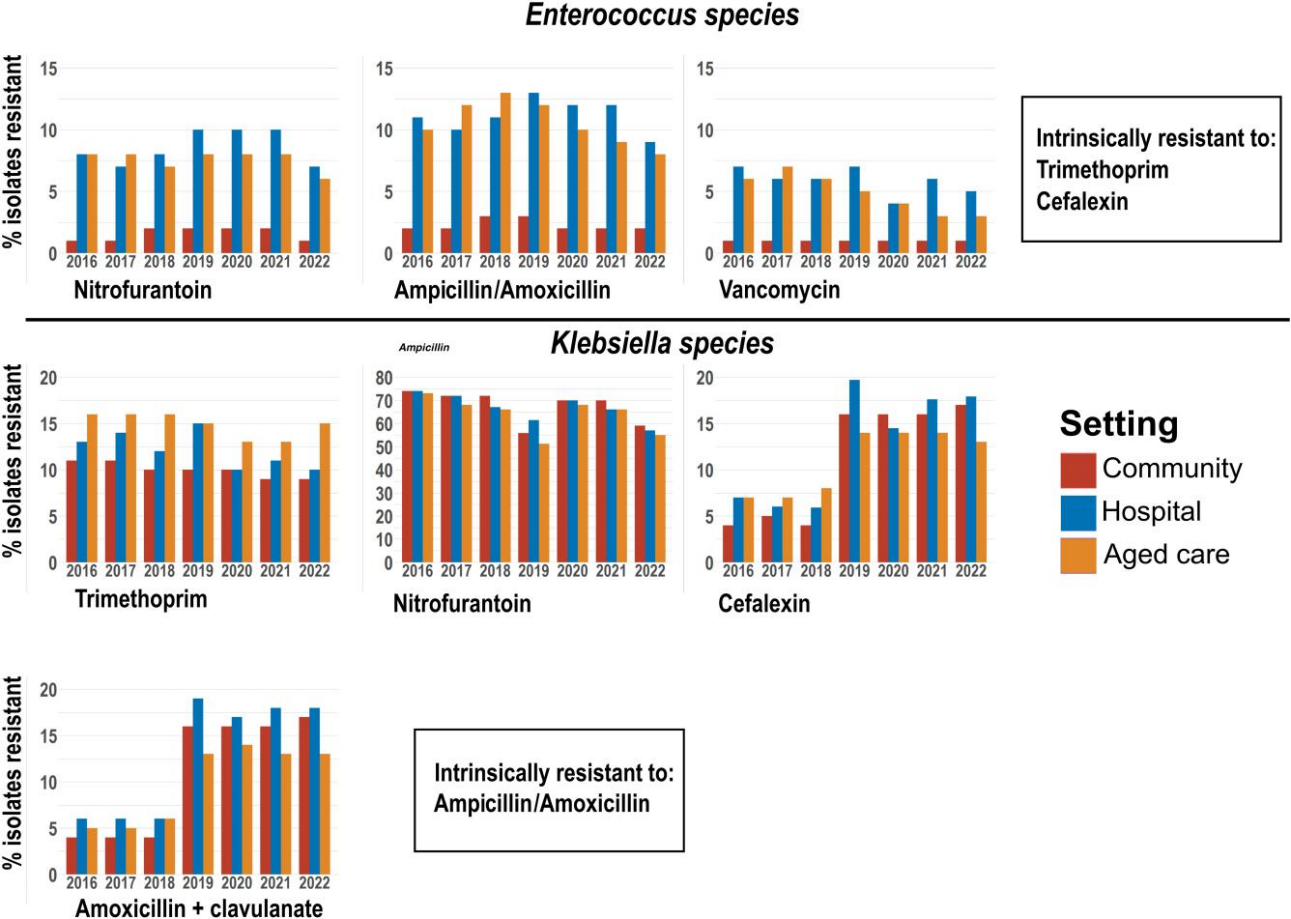
Resistance of *Enterococcus* and *Klebsiella* spp to antibiotics over time across clinical settings. Resistance is expressed as a percentage of the number of isolates tested within each setting, which may not represent the total number obtained. The counts for total individual species isolates obtained and the number tested for each antibiotic are shown in Supplementary Table 4.

Resistance to nitrofurantoin, ampicillin/amoxicillin, and vancomycin remained <15% across the 7-year period examined (Figure 2). The resistance rates of *Enterococcus* spp isolated from samples from hospitals and aged care facilities were significantly higher than that from the community (adjusted α = .0167, *P* < .001; Table 4). Ampicillin/amoxicillin resistance rose within all clinical settings in the prepandemic period (*P* < .001) before decreasing significantly from 2019 (*P* < .001). In aged care facilities, resistance to vancomycin decreased (*P* < .001).

#### *Klebsiella* Species

Trimethoprim resistance remained between 10% and 15% (Figure 2), with the odds of resistance among aged care isolates 1.57 times higher than the community and 1.24 times higher than hospitals (adjusted α = .0167, all *P* < .001; Table 4). Resistance to nitrofurantoin was similar between settings and consistently above 50%.

Resistance to cefalexin and amoxicillin with clavulanate increased notably between 2018 and 2019, coinciding with the reclassification of *Enterobacter aerogenes* to *Klebsiella aerogenes*.

#### *Enterobacter* Species

**Figure 3.**
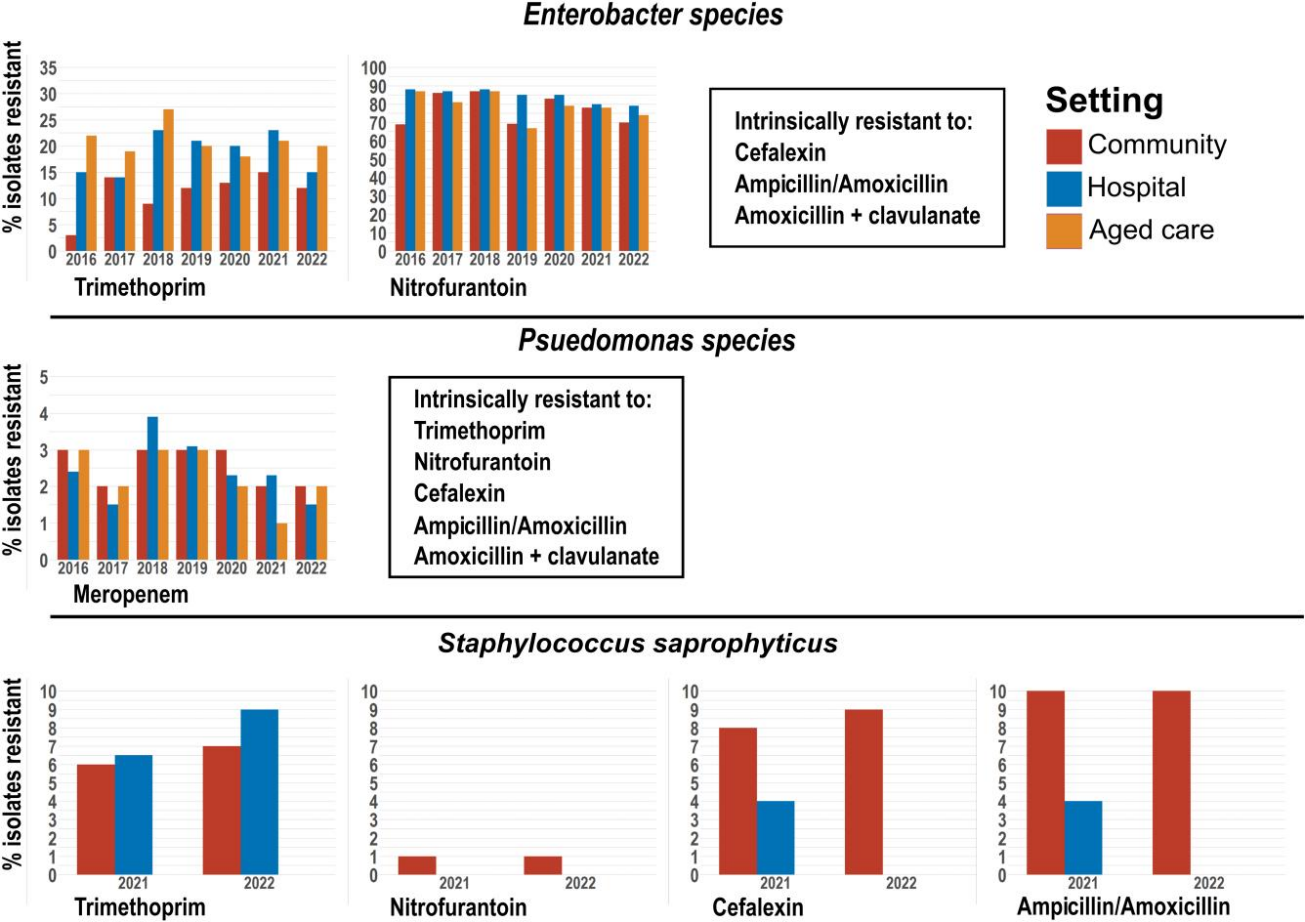
Resistance of *Enterobaccter* spp, *Pseudomonas* spp, and *Staphylococcus saprophyticus* to antibiotics over time across clinical settings. Resistance is expressed as a percentage of the number of isolates tested within each setting, which may not represent the total number obtained. The counts for total individual species isolates obtained and the number tested for each antibiotic are shown in Supplementary Table 4.

Trimethoprim resistance was notably lower in the community than in hospital and aged care settings, with yearly variations across settings (Figure 3, Table 4). Resistance to nitrofurantoin was very high (65%–90%).

#### *Pseudomonas* Species

Resistance to meropenem remained <5% throughout the period examined and tended to fluctuate 1%–2% between years, with no significant trends identified within or between settings (Figure 3). Susceptibilities for norfloxacin, gentamicin, and ceftazidime were not available for 2022, but similarly were 5% or less throughout 2016–2021 (Supplementary Figure 3).

#### Staphylococcus saprophyticus

Resistance to the 4 antibiotics tested remained <10%, with trimethoprim resistance highest within hospitals and all other antibiotics tested recording higher within the community (Figure 3).

### Predicted Resistance to Antibiotic Treatment for UTIs

Based on the antibiotic resistance patterns of the uropathogens *E coli*, *Enterococcus* spp, *Klebsiella* spp, *Enterobacter* spp, *Pseudomonas* spp, and *S saprophyticus* revealed in our analysis, we predicted the likelihood of resistance to the antibiotics recommended for treatment of UTIs in Australia. Before the COVID-19 pandemic years (2016–2019), 34.6%, 46.8%, and 44.4% of UTIs from the community, hospitals, and aged care would be resistant to trimethoprim as empirical therapy (Table 5). During the COVID-19 pandemic period (2020−2022), these figures were 30%, 43.6%, and 38.8%, respectively (Table 5). Both before and during the COVID-19 pandemic, less than 15% of UTIs from the community were resistant to nitrofurantoin or amoxicillin with clavulanate (Table 5). Between 24% and 28.5% of UTIs from hospitals and aged care facilities were resistant to nitrofurantoin and amoxicillin with clavulanate within this 7-year period (Table 5).

**Table 5.** Probability of Uropathogen Resistance to Antibiotics Recommended by the Australian Antibiotic Guideline for Treatment of Urinary Tract Infection

Antibiotic	2016–2019				2020–2022			
	Community	Hospital	Aged Care	All Settings	Community	Hospital	Aged Care	All Settings
Trimethoprim	0.346	0.468	0.444	0.380	0.300	0.426	0.388	0.335
Nitrofurantoin	0.149	0.271	0.244	0.174	0.146	0.260	0.241	0.169
Cefalexin	0.253	0.441	0.353	0.299	0.227	0.403	0.329	0.269
Amoxicillin plus clavulanate	0.125	0.282	0.221	0.163	0.136	0.223	0.223	0.172

Trimethoprim, nitrofurantoin, and cefalexin are recommended for treatment of cystitis. Amoxicillin plus clavulanate is recommended for treatment of acute nonsevere pyelonephritis.

## DISCUSSION

This study has evaluated 775 559 uropathogens and their resistance to antibiotics across the community, hospitals, and aged care facilities spanning the prepandemic period of 2016–2019 and 3 years from the emergence of COVID-19 in Australia, 2020–2022. Furthermore, we have predicted the probabilities of resistance of UTIs to the recommended antibiotics for treatment in Australia using a mathematical model developed in this study, based on both pathogen frequency and their antibiotic resistance rates.

Globally, 65%–75% of UTIs are caused by *E coli* [20], and similar prevalence was noted in our study (Table 1). Additionally, we observed that the causative pathogens for UTIs had clear associations with the clinical settings where samples had been obtained. *Escherichia coli* and *S saprophyticus* were associated with UTIs from the community while other bacterial species such as *Klebsiella*, *Pseudomonas*, and *Enterobacter* spp were more common in aged care facilities and hospitals (Table 2). Patients from hospitals and aged care facilities are more likely to have complicated UTIs such as catheter-associated UTIs, which are known to be associated with causative organisms such as *Klebsiella*, *Pseudomonas*, and *Enterobacter* spp in addition to *E coli* [21]. *Staphylococcus saprophyticus* is known to be an important uropathogen in sexually active young women and the second-most common cause of UTIs in females 16–25 years old [19, 22], which is consistent with our finding that this species was significantly more common in UTIs within the community when compared to hospitals and aged care facilities.

We examined the AMR patterns of the 6 most common uropathogens to the recommended empirical treatment for UTIs in Australia, including trimethoprim, cefalexin, nitrofurantoin, and amoxicillin with clavulanate [4] (Supplementary Table 1). Our data showed that the overall resistance of uropathogens to trimethoprim was high. *Escherichia coli* isolates had a resistance rate of 20%–30% depending on clinical settings (Figure 1). Consistent with our findings, a previous study conducted within northern Australia reported a 31% rate of trimethoprim resistance in uropathogenic *E coli* [6]. While resistance was <15% in *Enterobacter* spp isolated from the community, aged care facility and hospital isolates frequently recorded resistance rates of 20% or above (Figure 3). Among *Klebsiella* spp, resistance was much lower at approximately 15% (Figure 2). Both *Enterococcus* and *Pseudomonas* spp are intrinsically resistant to trimethoprim due to their ability to utilize folic acid from the environment (Figures 2 and 3).

Based on the data from last 3 years, our modeling has predicted that for empirical therapy, 30% of UTIs in the community, 42.6% in hospitals, and 38.8% in aged care facilities would be resistant to trimethoprim treatment (Table 5). Within the Enterobacterales, the *dfr* genes are almost exclusively responsible for trimethoprim resistance, with 95% of these genes located on plasmids [23, 24]. The collaboration between the European Society of Clinical Microbiology and Infectious Diseases and the Infectious Diseases Society of America identified rates of 20% resistance as the cutoff for effective trimethoprim empirical therapy [25]. In the United Kingdom and the Netherlands, similarly high trimethoprim resistance was addressed by changing the suggested therapy to nitrofurantoin [26]. Studies since have identified an inverse association between nitrofurantoin use and trimethoprim resistance [26, 27]. Although the current Australian Therapeutic Guidelines continue to recommend trimethoprim due to the low risk of adverse outcomes from treatment failure [4], our data suggest a necessary evaluation of trimethoprim as the first-line therapy for empirical treatment of cystitis. A recent Australian study identified that in general practice 72% of antibiotic prescriptions for UTI were accompanied by susceptibility testing [28], indicating that our antibiogram likely reflects the broader resistance patterns present in the community.

The overall resistance of uropathogens in our study to cefalexin was moderate, and that of nitrofurantoin and amoxicillin with clavulanate was low, except for in those with intrinsic resistance mechanisms (Figures 1–3). Our modeling has predicted that for empirical therapy, <15% of UTIs in the community, <25% of UTIs in aged care, and <27.5% of UTIs from hospitals were resistant to nitrofurantoin (Table 5). This suggests that nitrofurantoin is more appropriate than trimethoprim as the first-line empirical therapy for treatment of cystitis in the Australian population. The resistance of UTIs to amoxicillin with clavulanate was slightly lower than nitrofurantoin, while cefalexin resistance was moderate (Table 5).

While AMR rates currently suggest cefalexin, amoxicillin with clavulanate and nitrofurantoin remain appropriate for empirical therapy, nitrofurantoin remains highly important, and both amoxicillin with clavulanate and cefalexin are ranked at medium importance within the Australian ratings [29]. Conversely, trimethoprim is ranked low. This should be considered alongside AMR trends and the risks of proliferation of the resistance reservoir through co-selection when determining which changes to Australian empirical UTI therapy may be appropriate.

Bacterial pathogens isolated from hospitals are known to have a higher resistance to antibiotics. Interestingly, we found that the resistance rates of *E coli* to trimethoprim and cefalexin were the highest within the aged care facilities (Figure 1, Table 4). A previous study from the Netherlands reported that nursing homes have the potential to drive and sustain AMR epidemics within the healthcare system [30]. The high transmission of AMR in nursing homes was largely attributed to the longer length of patients in these facilities [30]. Our findings suggest that it is important to consider aged care facilities in surveillance and monitoring the spread of AMR in Australia.

During the 3 years of the COVID-19 pandemic in Australia from 2020 to 2022, there was a trend of decreased resistance of *E coli* to trimethoprim and cefalexin (Figure 1) and *Enterococcus* spp to ampicillin/amoxicillin and vancomycin in aged care facilities (Figure 2). Although patient interactions with the healthcare system undoubtedly varied throughout this time, the number of uropathogen isolations obtained by DHM fell only temporarily in 2020, before increasing once more in 2021 and 2022. Additionally, it does not seem that the incidence of UTIs changed substantially with the pandemic, with an Australian study noting no discernible changes in trimethoprim prescribing from 2019 to 2020 [31]. While the reasons behind these changes are not clear, they may represent a compelling case for the effect of the enhanced infection control procedures maintained following the emergence of COVID-19 in reducing the spread of antibiotic resistance.

Our analysis has demonstrated that UTIs in the community, hospitals, and aged care facilities differ in uropathogens and AMR, further emphasizing the necessity for sensitivity testing to inform treatment and for continued global surveillance. We also showed that uropathogen resistance is high to trimethoprim, but low to amoxicillin with clavulanate and nitrofurantoin. Our data provide valuable information to medical professionals and to relevant authorities in formulating policies for empirical antibiotic therapy of UTIs in Australia.

## Supplementary Material

ofad676_Supplementary_Data

## Supplementary Data

Supplementary materials are available at *Open Forum Infectious Diseases* online. Consisting of data provided by the authors to benefit the reader, the posted materials are not copyedited and are the sole responsibility of the authors, so questions or comments should be addressed to the corresponding author.

## Notes

***Author contributions.*** A. M. Y. conducted data analysis. M. M. T. calculated antibiotic resistance probabilities. C. Y. helped with the organization of the patient data. M. C. W. organized and provided uropathogen and antibiotic data used for analysis in this study. L. Z. initiated and supervised the project. A. M. Y. wrote the first version of the manuscript. All authors contributed to manuscript revision.

***Acknowledgments.*** We thank Nancy Briggs and Zhixin Liu, statistical consultants at Stats Central, Mark Wainwright Analytical Centre, University of New South Wales, for their advice on statistical analysis. A. M. Y. and C. Y. acknowledge support from the Australian Government Research Training Program Scholarship.

***Patient consent*.** The use of aggregated bacterial isolation data provided by the Douglass Hanly Moir Pathology diagnostic laboratory for this retrospective study was approved by the University of New South Wales Human Research Advisory Panel executive (HC200755). This project did not include factors necessitating patient consent.

***Data availability.*** Data are available in the Supplementary Materials.

***Financial support.*** This work was supported by a Faculty Research Grant (grant number PS46772) awarded to L. Z. from the University of New South Wales.
