# Supplementary material for: Clinical Setting Comparative Analysis of Uropathogens and Antibiotic Resistance: A Retrospective Study Spanning the Coronavirus Disease 2019 Pandemic
Source: Open Forum Infect Dis. 2023 Dec 22;11(2):ofad676. doi: 10.1093/ofid/ofad676 (PMC10853000; doi:10.1093/ofid/ofad676)
Supplement: ofad676_Supplementary_Data [file ofad676_supplementary_data.pdf]

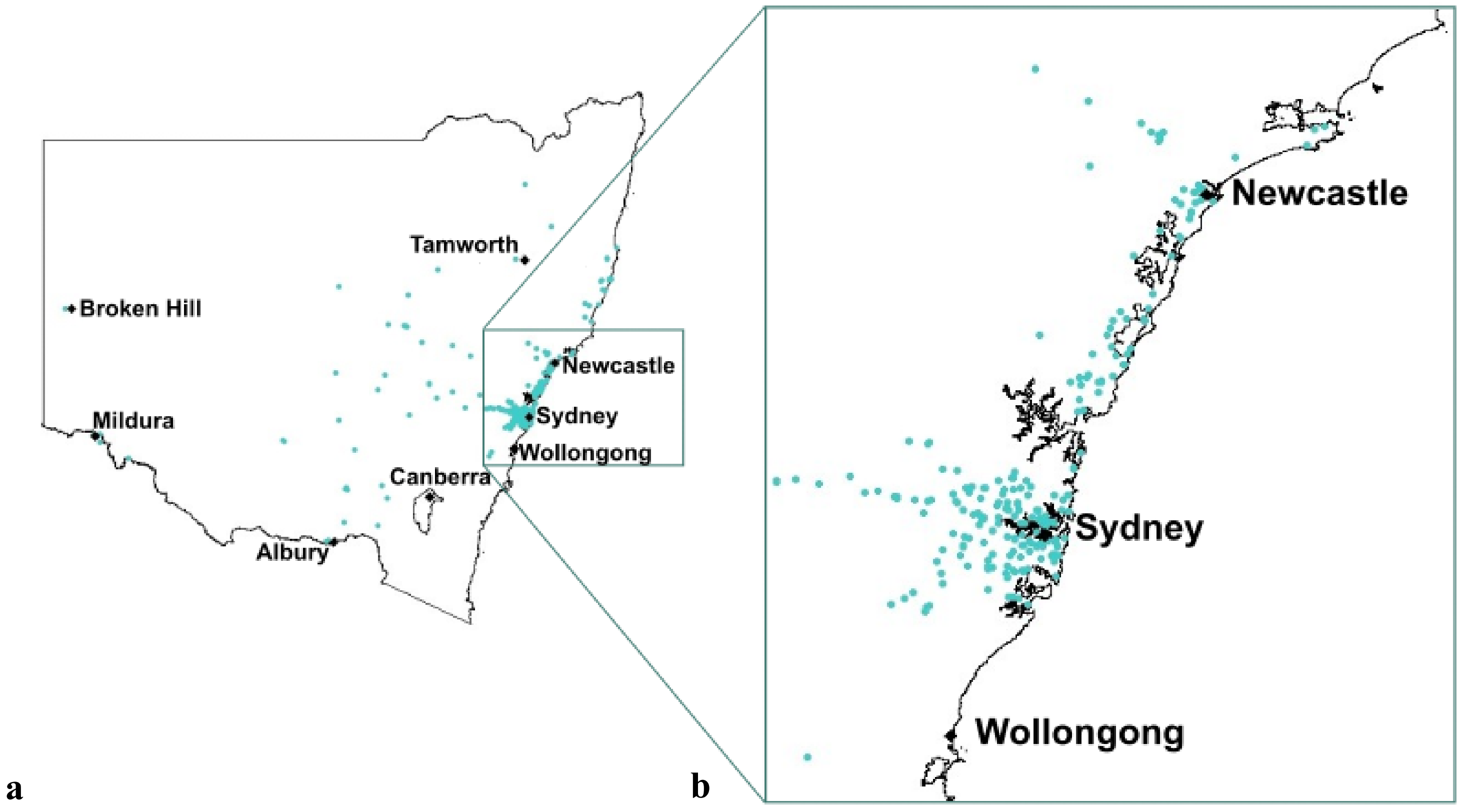

Supplementary Figure 1 Map of DHM pathology collection locations within the state of New South Wales, Australia. Each location is represented by a single point. **a** Map of the state of NSW depicting the location of all 323 collection sites. **b** The majority of collection sites (74%, 238/323) were located in the 186km metropolitan region between the cities of Wollongong and Newcastle.

Supplementary Table 1 Uropathogens contributing to the all other isolates category obtained from patients with UTI in the years 2016-2022 by clinical setting.

Setting	Organism Group	Number of isolates for individual uropathogen							
		2016	2017	2018	2019	2020	2021	2022	Total
Community	Other Enterobacterales	336	324	7398	7407	NA	NA	NA	15465
	Coagulase negative *Staphylococci*	4872	4588	NA	NA	NA	NA	NA	9460
	Staphylococcus aureus	903	818	782	720	709	746	621	5299
	Citrobacter spp.	2399	2638	NA	NA	NA	NA	NA	5037
	Morganella morganii	1253	1270	NA	NA	NA	NA	NA	2523
	ß haemolytic *Streptococci*	395	374	327	267	321	284	118	2086
	Viridans *Streptococci*	703	611	NA	NA	NA	NA	NA	1314
	Serratia marcescens	465	440	NA	NA	NA	NA	NA	905
	Providencia rettgeri	69	69	70	74	81	0	NA	363
	Acinetobacter baumanii	108	108	NA	NA	NA	NA	NA	216
	Corynebacterium spp.	61	58	NA	NA	NA	NA	NA	119
	Haemophilus influenzae	NA	40	35	NA	NA	NA	NA	75
	Fastidious Gram-negative bacteria	NA	31	NA	NA	NA	NA	NA	31
	Below reportable cutoff	121	42	5	42	20	30	14	274
Hospital	Other Enterobacterales	NA	35	860	839	NA	NA	NA	1734
	Staphylococcus aureus	116	96	124	92	97	91	88	704
	Coagulase negative *Staphylococci*	213	226	NA	NA	NA	NA	NA	439
	Citrobacter spp.	233	196	NA	NA	NA	NA	NA	429
	Morganella morganii	147	172	NA	NA	NA	NA	NA	319
	ß haemolytic *Streptococci*	79	90	71	36	31	NA	NA	307
	Serratia marcescens	68	84	NA	NA	NA	NA	NA	152
	Viridans *Streptococci*	62	57	NA	NA	NA	NA	NA	119
	Below reportable cutoff	98	125	17	15	29	24	13	321
Aged care	Other Enterobacterales	34	33	1307	1320	N	NA	NA	2694
	Staphylococcus aureus	185	204	184	171	207	216	157	1324
	Citrobacter spp.	282	252	NA	NA	NA	NA	NA	534
	Viridans *Streptococci*	137	122	NA	NA	NA	NA	NA	259
	Morganella morganii	121	114	NA	NA	NA	NA	NA	235
	Coagulase negative *Staphylococci*	86	87	NA	NA	NA	NA	NA	173
	ß haemolytic *Streptococci*	40	38	54	NA	NA	NA	NA	132
	Serratia marcescens	41	44	NA	NA	NA	NA	NA	85
	Below reportable cutoff	117	126	44	54	63	34	13	451

The below reportable cutoff group consists of bacterial isolates of an organism group which were obtained ≤ 30 times within a clinical setting in the given year.

Supplementary Table 2 χ^2 comparison of uropathogen isolation between 2016-2022 by clinical setting.

Organism	Setting		
	**Community N (%)**	**Hospital N (%)**	**Aged Care N (%)**
E. coli	426995 (66.3) [-91]	27851 (48.8) [-78.5]	41628 (56.2) [-46.3]
***Enterococcus* species**	74902 (11.6) [-23.2]	10092 (17.7) [-43.4]	8136 (11) [-8.9]
***Klebsiella* species**	53034 (8.2) [-33.1]	6021 (10.6) [-16.3]	8471 (11.4) [-27.8]
***Pseudomonas* species**	18267 (2.8) [-102.1]	5435 (9.5) [-73.5]	6065 (8.2) [-64.9]
***Enterobacter* species**	12196 (1.9) [-30.3]	2043 (3.6) [-25.3]	2169 (2.9) [-16.2]
S. saprophyticus	7126 (1.1) [-36.1]	74 (0.1) [-20.7]	0 (0) [-27.7]
**All other isolates**	51990 (8.1) [-22.6]	5522 (9.7) [-11.6]	7542 (10.3) [-18.6]
**$X^2(12)$**	18872		
**Cramer's V**	0.110 (CI 0.109 - 0.112)		

Adjusted standardised residuals appear in parenthesis below the isolation frequencies. All adjusted residuals were individually consistent with a significant association between clinical setting and incidence of species isolation ($P < 0.001$).

Supplementary Table 3 Indications associated with urinary tract infections and recommended antibiotic treatment in adults as described in the Australian Therapeutic Guidelines 2021 [4].

**Indication**	**Delivery**	**Empirical therapy**	**Second line and/or directed therapy**
Acute cystitis	Oral	Trimethoprim<br>Nitrofurantoin<br>*Cefalexin*	Amoxicillin<br>Trimethoprim + sulfamethoxazole<br>Amoxicillin + clavulanate<br>Fosfomycin<br>Norfloxacin<br>Ciprofloxacin
Acute non-severe pyelonephritis	Oral	Amoxicillin + clavulanate<br>*Ciprofloxacin*	Amoxicillin<br>Trimethoprim<br>Cefalexin<br>Trimethoprim + sulfamethoxazole<br>Ciprofloxacin
Severe pyelonephritis	IV	Gentamicin + Amoxicillin/Ampicillin<br>*Cefotaxime/Ceftriaxone*	As for non-severe pyelonephritis
Sepsis from urinary tract source	IV	Gentamicin + Amoxicillin/Ampicillin<br>*Cefotaxime/Ceftriaxone*	Meropenem

Alternatives when first line therapies cannot be used due to allergy or contraindication are italicised.

Supplementary Table 4 Number of urine specimen isolates assessed for resistance by antibiotic by clinical setting.

Isolate	Source	Antibiotic	2016	2017	2018	2019	2020	2021	2022
E. coli									
	Community	Total isolates obtained	66001	66172	60999	61327	56628	54592	61276
		Ampicillin/Amoxicillin	65997	66164	60994	61319	56617	54574	61261
		Cefalexin	65992	66159	58863	61318	56420	54583	61268
		Nitrofurantoin	65996	66165	59975	51945*	56617	54587	61260
		Trimethoprim	65973	66145	60975	61306	56599	54284	61234
		Norfloxacin[#]	65996	38527*	20527*	61090	6732*	6307*	NA
		Cefotaxime/Ceftriaxone[#]	4206*	4541*	4126*	4673*	3881*	NA	NA
		Gentamicin[#]	4378*	4665*	4402*	4698*	4207*	3922*	NA
		Meropenem[#]	4213*	4547*	4334*	4677*	3879*	3544*	NA
	Hospital	Total isolates obtained	3977	4314	3967	3655	3451	3722	4765
		Ampicillin/Amoxicillin	3976	4314	3965	3655	3451	3721	4765
		Cefalexin	3949	4303	3271*	3066*	2876*	3113*	4124
		Nitrofurantoin	3976	4313	3913	3285*	3451	3722	4764
		Trimethoprim	3976	4310	3965	3655	3450	3710	4764
		Norfloxacin[#]	3961	2606*	1795*	3579	615*	653*	NA
		Cefotaxime/Ceftriaxone[#]	2584*	2725*	2488*	2268*	384*	NA	NA
		Gentamicin[#]	3311*	3586*	3379*	2966*	2670*	3051*	3953
		Meropenem[#]	2595*	2770*	2621*	2328*	422*	430*	NA
	Aged care	Total isolates obtained	5505	5268	5360	5382	6132	6754	7227
		Ampicillin/Amoxicillin	5505	5268	5359	5381	6132	6753	7226
		Cefalexin	5504	5267	5164	5381	6101	6753	7226
		Nitrofurantoin	5505	5266	5284	4524*	6131	6754	7226
		Trimethoprim	5504	5266	5358	5381	6130	6715	7221
		Norfloxacin[#]	5504	3113*	1737*	5366	1234*	1320*	NA
		Cefotaxime/Ceftriaxone[#]	618*	544*	642*	651*	821*	NA	NA
		Gentamicin[#]	621*	545*	686*	655*	844*	901*	NA
		Meropenem[#]	620*	544*	681*	655*	817*	878*	NA
Enterococcus species									
	Community	Total isolates obtained	16439	14944	9085	8760	8453	8870	8351
		Ampicillin/Amoxicillin	16432	14941	9079	8757	8446	8865	8350
		Nitrofurantoin	16430	14939	9073	8704	8452	8869	8349
		Vancomycin	16425	14933	9069	8758	8448	8867	8345
	Hospital	Total isolates obtained	1913	1871	1341	1341	1216	1242	1277
		Ampicillin/Amoxicillin	1910	1869	1341	1232	1216	1240	1277
		Nitrofurantoin	1911	1865	1338	1218	1216	1239	1276
		Vancomycin	1911	1870	1340	1231	1215	1241	1277
	Aged care	Total isolates obtained	1272	1222	1101	1004	1185	1403	949

		Ampicillin/Amoxicillin	1271	1222	1101	1004	1185	1403	948
		Nitrofurantoin	1272	1221	1101	997	1184	1403	949
		Vancomycin	1272	1222	1099	1003	1184	1401	949
Klebsiella species									
	Community	Total isolates obtained	7388	7519	6956	7856	7521	7172	8622
		Cefalexin	7385	7514	6712	7854	7504	7166	8611
		Nitrofurantoin	7385	7517	6837	6544*	7516*	7167	8613
		Trimethoprim	7382	7511	6946	7843	7508	7135	8611
		Norfloxacin▪	7384	4545*	2248*	7814	531*	354*	NA
		Ceftotaxime/Ceftriaxone▪	702*	759*	666*	808*	368*	NA	NA
		Gentamicin▪	738*	781*	709*	807*	463*	450*	NA
		Meropenem▪	703*	760*	694*	806*	349*	324*	NA
	Hospital	Total isolates obtained	716	830	790	862	799	884	1140
		Cefalexin	712	827	673*	739*	685*	741*	1019
		Nitrofurantoin	716	829	777	768*	799	882	1137
		Trimethoprim	716	829	789	861	798	878	1140
		Norfloxacin▪	715	535*	344*	837	114*	89*	NA
		Ceftotaxime/Ceftriaxone▪	505*	549*	520*	581*	82*	NA	NA
		Gentamicin▪	620*	703*	660*	724*	609*	727*	981
		Meropenem▪	506*	551*	533*	586*	87*	72*	NA
	Aged care	Total isolates obtained	1020	921	969	1113	1281	1510	1657
		Cefalexin	1020	921	930	1113	1278	1510	1657
		Nitrofurantoin	1020	920	952	935*	1280	1509	1656
		Trimethoprim	1019	920	966	1111	1280	1501	1656
		Norfloxacin▪	1020	547*	287*	1110	139*	131*	NA
		Ceftotaxime/Ceftriaxone▪	91*	72*	98*	97*	92*	NA	NA
		Gentamicin▪	93*	72*	100*	98*	96*	131*	NA
		Meropenem▪	92*	72*	100*	98*	87*	122*	NA
Enterobacter species									
	Community	Total isolates obtained	2477	2465	2211	1259	1282	1170	1332
		Nitrofurantoin	2476	2464	2165	1063*	1280	1168	1330
		Trimethoprim	2477	2464	2210	1255	1280	1161	1332
		Norfloxacin▪	2476	156*	705*	1247	270*	205*	NA
		Gentamicin▪	388*	424*	365*	279*	235*	231*	NA
		Meropenem▪	371*	416*	360*	277*	168*	142*	NA
	Hospital	Total isolates obtained	344	379	345	234	256	226	259
		Nitrofurantoin	344	379	343	211	255	224	259
		Trimethoprim	344	378	345	234	256	224	259
		Norfloxacin▪	344	239*	162*	228	119*	96*	NA
		Gentamicin▪	304*	320*	307*	202*	217*	202*	239

		Meropenem▪	249*	288*	285*	195*	113*	96*	NA
	Aged care	Total isolates obtained	367	310	338	223	296	314	321
		Nitrofurantoin	365	310	332	188*	296	314	321
		Trimethoprim	365	310	337	223	296	313	320
		Norfloxacin▪	365	188*	101*	221	62*	74*	NA
		Gentamicin▪	78*	64*	95*	47*	61*	68*	NA
		Meropenem▪	77*	64*	93*	48*	38*	49*	NA
Pseudomonas species									
	Community	Total isolates obtained	2779	2792	2606	2685	2812	2542	2051
		Meropenem	2759	2781	2601	2674	2801	877*	2048
		Norfloxacin▪	2774	1680*	847*	2677	2802	839*	NA
		Ceftazidime▪	2750	2478*	2547	2658	2793	877*	NA
		Gentamicin▪	2775	2789	2602	2680	2808	803*	NA
	Hospital	Total isolates obtained	780	851	790	800	742	783	689
		Meropenem	636*	650*	616*	634*	572*	605*	521
		Norfloxacin▪	777	556*	388*	795	733	375*	NA
		Ceftazidime▪	634*	590*	688*	793	732	396*	NA
		Gentamicin▪	780	851	789	800	740	356*	NA
	Aged care	Total isolates obtained	886	873	855	872	948	879	752
		Meropenem	884	871	854	867	939	875	752
		Norfloxacin▪	886	515*	283*	871	946	260*	NA
		Ceftazidime▪	880	780*	838	868	941	271*	NA
		Gentamicin▪	884	872	855	870	944	247*	NA
S. saprophyticus									
	Community	Total isolates obtained	NA	NA	NA	NA	NA	3634	3492
		Trimethoprim	NA	NA	NA	NA	NA	3615	3491
		Nitrofurantoin	NA	NA	NA	NA	NA	3632	3492
		Cefalexin	NA	NA	NA	NA	NA	3632	3491
		Ampicillin/Amoxicillin	NA	NA	NA	NA	NA	3633	3492
	Hospital	Total isolates obtained	NA	NA	NA	NA	NA	31	43
		Trimethoprim	NA	NA	NA	NA	NA	24*	35
		Nitrofurantoin	NA	NA	NA	NA	NA	24*	35
		Cefalexin	NA	NA	NA	NA	NA	31	43
		Ampicillin/Amoxicillin	NA	NA	NA	NA	NA	31	43
	Aged care	Total isolates obtained	NA	NA	NA	NA	NA	0	0

An * indicates <90% of the total isolates obtained were tested for resistance, # indicates resistance rates shown in supplementary figure 2, ▪ indicates resistance rates shown in supplementary figure 3.

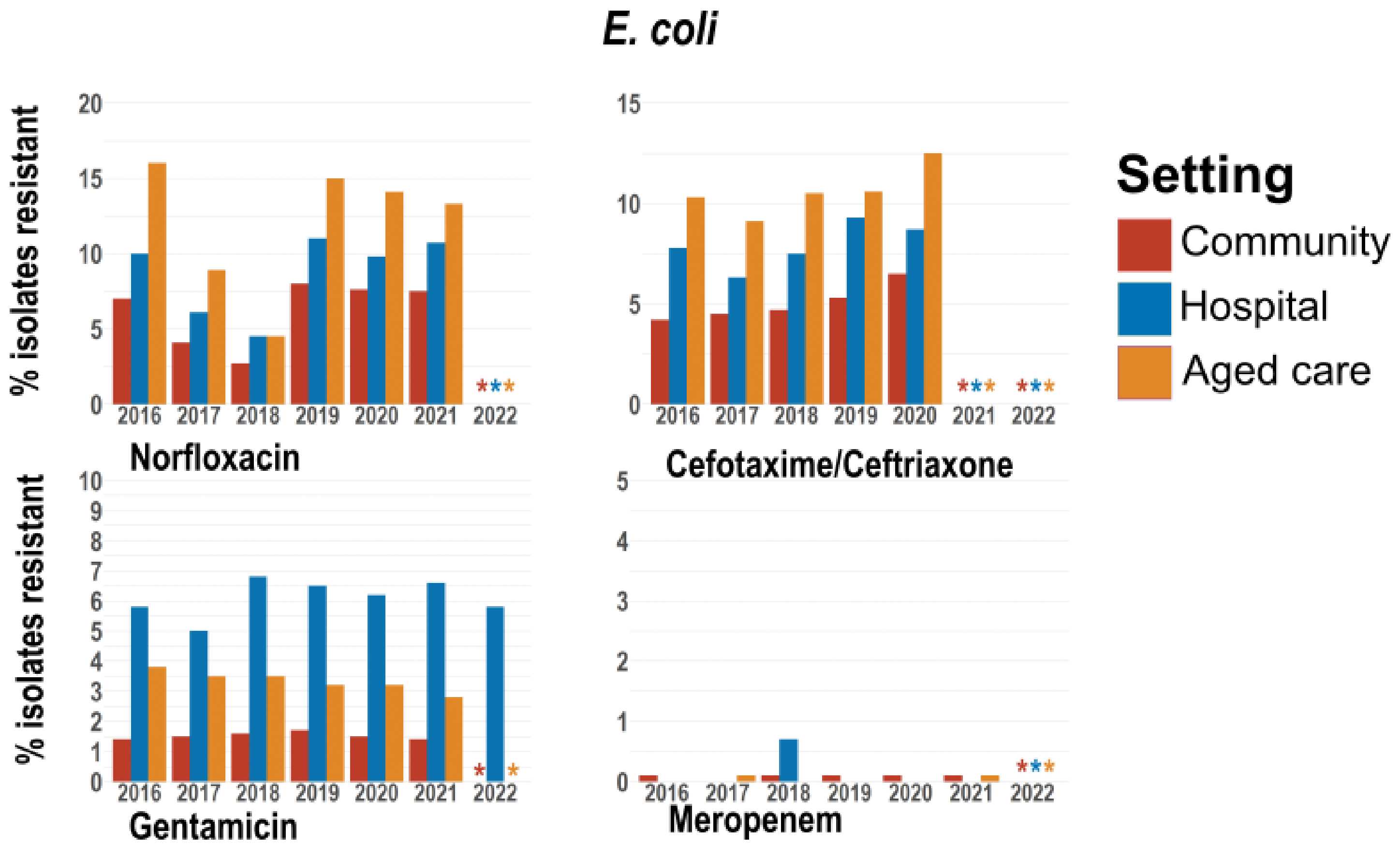

Supplementary Figure 2 Resistance of *E. coli* to antibiotics over time across clinical settings. Resistance is expressed as a percentage of the number of isolates tested within each setting, which may not represent the total number obtained. The counts for total individual species isolates obtained and the number tested for each antibiotic are shown in Supplemetary Table 4. An * indicates resistance data not available in that setting for that year.

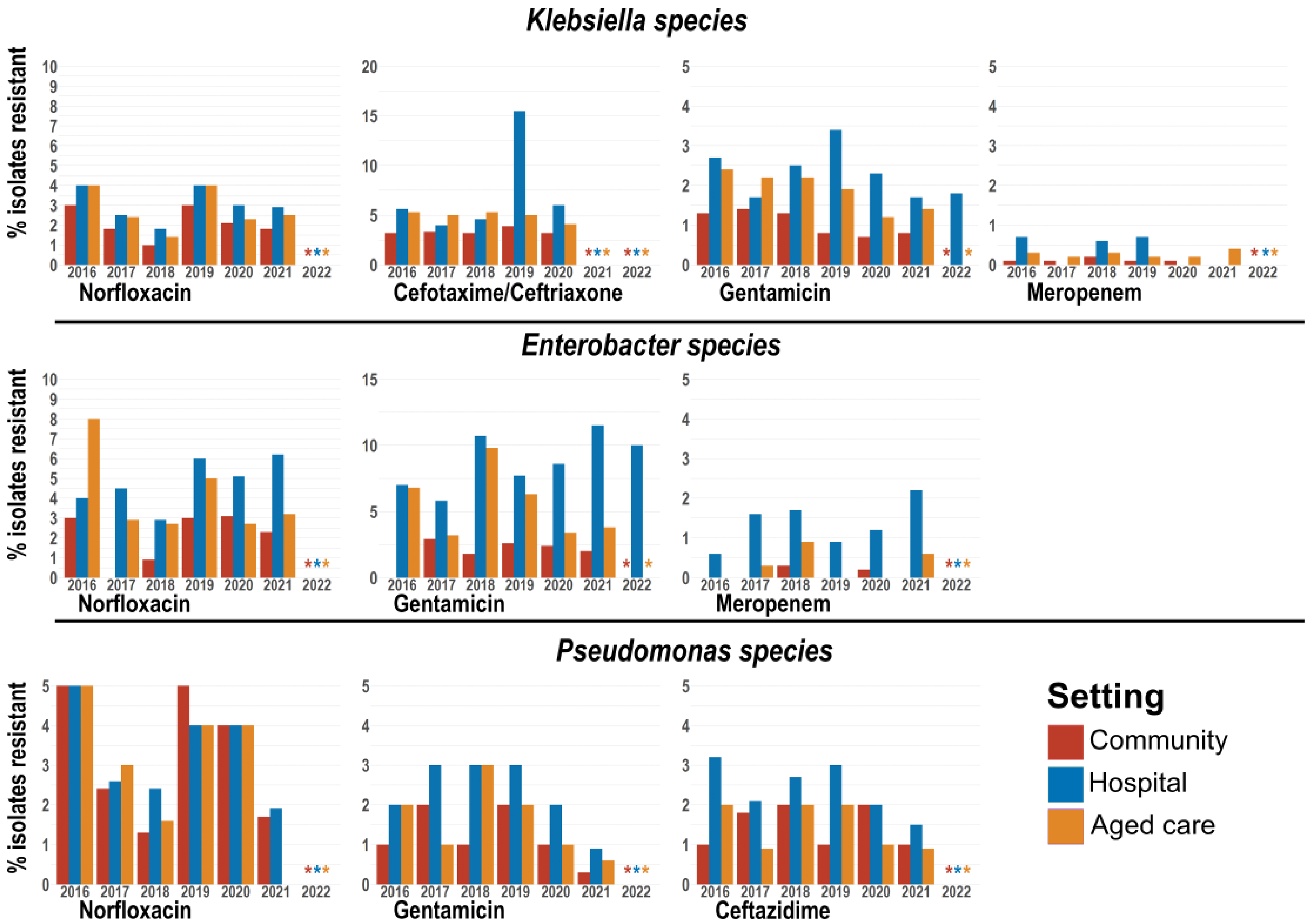

Supplementary Figure 3 Resistance of *Klebsiella* species, *Enterobacter* species and *Pseudomonas* species to antibiotics over time across clinical settings. Resistance is expressed as a percentage of the number of isolates tested within each setting, which may not represent the total number obtained. The counts for total individual species isolates obtained and the number tested for each antibiotic are shown in Supplemetary Table 4. An * indicates resistance data not available in that setting for that year.
